# The relationship between democracy and corruption and the global physician workforce

**DOI:** 10.1371/journal.pgph.0003656

**Published:** 2024-11-27

**Authors:** Amrit Kirpalani, Eray Yilmaz

**Affiliations:** 1 Department of Paediatrics, Schulich School of Medicine and Dentistry, Western University, London, Ontario, Canada; 2 Lawson Health Research Institute, London, Ontario, Canada; Aga Khan University, PAKISTAN

## Abstract

**Background:**

Understanding how governance factors such as democracy and corruption impact the healthcare workforce is crucial for achieving Universal Health Coverage (UHC). Effective health workforce planning and resource allocation are influenced by these political constructs. This study examines the relationship between democracy and corruption and key healthcare workforce metrics.

**Methods:**

A cross-sectional study was conducted using a global dataset from 2020 to 2022. The primary outcome was Physician Density (medical doctors per 10000 people). Secondary outcomes included the generalist to specialist ratio and the percentage of female physicians (% Female). Partial correlations, multivariate analysis of variance (MANOVA), and univariate analysis of variance (ANOVA) were used to analyze the relationship between workforce variables and the democracy index (DI), and corruption perception index (CPI), controlling for domestic health expenditure.

**Results:**

Data from 134 countries showed significant positive associations between both DI (r = 0.32, p = 0.004) and CPI (r = 0.43, p < 0.001) with physician density. MANOVA indicated significant multivariate effects of DI (Wilks’ Lambda = 0.8642, p = 0.013) and CPI (Wilks’ Lambda = 0.8036, p = 0.001) on the combined workforce variables. Univariate ANOVAs showed that DI (F = 6.13, p = 0.015) and CPI (F = 10.57, p = 0.002) significantly affected physician density, even after adjusting for domestic expenditure (F = 18.53, p < 0.001). However, neither DI nor CPI significantly impacted the Generalist to Specialist Ratio or % Female Physicians.

**Discussion:**

Higher levels of democracy and lower levels of corruption are associated with a greater density of medical doctors, independent of healthcare spending. Policymakers must advocate for governance reforms that support a robust healthcare workforce to support aim of universal health coverage.

## Introduction

Achieving Universal Health Coverage (UHC) is a pivotal goal for global health, and a robust healthcare workforce is essential for reaching this objective. The World Health Organization (WHO) has emphasized that addressing healthcare workforce issues is a "critical pathway" towards UHC, highlighting the importance of sufficient numbers of well-trained, equitably distributed healthcare professionals worldwide [1]. Despite substantial efforts from medical schools and academic societies to address the physician pipeline [2, 3], the impact of broader political factors on the healthcare workforce remains unclear. The distribution of healthcare resources is heavily influenced by political factors, which in turn shape the availability and effectiveness of healthcare services. The political dimensions of medical education and healthcare workforce distribution are intricately linked to broader global power dynamics.

The nature and stability of governance play critical roles in shaping policies that determine the distribution, training, and retention of healthcare professionals. These systems create the framework within which health policies are formulated, resources are allocated, and institutional trust is either fostered or eroded. However, governance indicators such as democracy and corruption are exogenous variables, and the relationship between governance and health workforce outcomes is not strictly unidirectional. In some cases, a robust healthcare system may itself contribute to improving governance outcomes by building public trust and institutional capacity. Conversely, good governance—through transparency, accountability, and effective policy-making—can enhance the healthcare workforce. Additionally, a country with lower governance rankings may still achieve workforce success through strategic national health programs, international collaborations, or the mobilization of local resources. This highlights that health workforce outcomes are also shaped by unmeasured factors such as economic development, regional stability, and migration-related push-pull dynamics, which influence both healthcare systems’ internal resilience and broader governance outcomes. As such, the impact of political systems on the healthcare workforce is not straightforward but rather a complex interaction of governance, policy-making, and the broader sociopolitical environment. Systems of governance, such as democracy, may also influence the healthcare workforce through the mechanisms of transparency, accountability, and public participation. Democracies, characterized by these features, often promote better health outcomes by ensuring that resources are allocated in a manner that reflects public needs [4]. For instance, in democratic nations the political stability and institutional integrity provided by the governance structure can lead to better investment in healthcare infrastructure, and countries are more likely to invest in public health infrastructure, health education, and preventive care, which collectively contribute to better health outcomes and reduced health disparities [5].

However, the influence of political systems on the healthcare workforce is not limited to the type of governance alone; as noted previously, power relations within and across these systems also play a significant role [6]. Corruption, as a manifestation of power abuse, can undermine even the most well-structured governance systems by diverting resources, fostering inequality, and eroding public trust. Unlike democracy, which provides a framework for governance, corruption is a phenomenon that can occur within any political system, be it democratic, autocratic, or otherwise [7]. It represents the failure of governance to protect public resources and ensure their equitable distribution. In healthcare, corruption in governance can lead to the misallocation of funds, reduced investment in essential services, and the degradation of working conditions for healthcare professionals, ultimately weakening the entire healthcare workforce [8].

Understanding how these political constructs relate to the global physician workforce is critical, as governmental policies and the overall quality of governance can profoundly affect workforce distribution and resource allocation within healthcare systems.

This study investigates the relationship between the global impact of democracy and corruption on key healthcare workforce metrics, with the goal of supporting health systems leaders in bridging the influence of institutional efforts with broader governmental actions. The present study draws upon political stability theories and resource allocation models in healthcare [9].

## Methods

This was a cross-sectional study designed to examine the relationship between democracy and corruption on key healthcare workforce metrics across a global dataset. Data were collected from multiple reputable sources, including the World Health Organization (WHO), Economist Intelligence Unit (EIU), and Transparency International. The study period covers data from 2020 to 2022, ensuring a contemporary analysis of the relationships between governance indicators and healthcare workforce metrics, moreover this timeline includes the most recent data for the majority of countries in the WHO data repository.

### Data sources

- **Democracy Index (DI):** The Democracy Index, compiled by the Economist Intelligence Unit, scores countries on a scale from 0 to 10. It assesses five categories: civil liberties, political culture, political participation, functioning of government, and electoral process. These scores provide a comprehensive measure of the level of democracy in each country [10].
- **Corruption Perception Index (CPI)**: The Corruption Perception Index, compiled by Transparency International, scores countries on a scale from 0 to 100, with *higher* scores indicating *lower* levels of perceived corruption. The CPI aggregates data from various sources, including expert assessments and opinion surveys, to provide a robust measure of public sector corruption [11].
- **Healthcare Workforce Metrics**: Data on the number of medical doctors per 10,000 population (Physician density), the generalist to specialist ratio, and the percentage of female physicians were sourced from the WHO’s Global Health Workforce Statistics database. These metrics offer insights into the composition and distribution of the healthcare workforce in each country [12].
- **Domestic Health Expenditure**: The proportion of a country’s Gross Domestic Product (GDP) allocated to health expenditure was also sourced from the WHO. This variable was used as a control to account for the economic capacity of each country to support its healthcare system [1].

### Sampling and categorization

Countries were categorized into "High Democratic" and "Low Democratic" based on whether their DI score was above or below the median DI score of the sample. Similarly, countries were classified into "Low Corruption" and "High Corruption" groups based on their CPI scores relative to the median CPI score. This binary categorization, based on author consensus, facilitated the analysis of the impact of varying levels of democracy and corruption on healthcare workforce metrics. The decision to use binary categorization was made to emphasize the clearest differences in governance quality across countries, allowing for clearer identification of the relationship between governance and healthcare workforce outcomes.

### Statistical analysis

Descriptive statistics, including means, standard deviations, medians, and interquartile ranges, were calculated for all variables. Partial correlations between DI/CPI and each dependent variable (physician density, generalist to specialist ratio, % female physicians) were computed while controlling for domestic health expenditure. Multivariate Analysis of Variance (MANOVA) was conducted to assess the combined impact of DI and CPI on the dependent workforce variables and Univariate ANOVAs were performed to evaluate the individual effects of DI and CPI on each dependent variable, with domestic expenditure included as a confounder. The statistical significance threshold was set at p < 0.05. All statistical analyses were performed using R (version 4.1.2) and SPSS (version 27).

### Ethical considerations

This study was exempt from ethics board approval in accordance with the Tri-Council Policy Statement Article 2.2, as it utilized publicly available, de-identified data. No direct interaction with human subjects was involved, and there were no risks posed to individuals or communities.

## Results

A total of 134 countries were included in the analysis, representing a diverse range of governance structures and healthcare systems. The descriptive statistics for the main variables of interest are summarized in Table 1. The mean Democracy Index (DI) score was 5.611 (SD = 2.099), with scores ranging from 0.32 to 9.93. The mean Corruption Perception Index (CPI) score was 41.10 (SD = 20.26), with a range of 18 to 88. After grouping countries there were 67 countries in each of the ‘High’ and ‘Low’ DI and CPI groups respectively. The average physician density (MD per 10,000 people) was 24.45 (SD = 14.32), ranging from 2.54 to 58.58. The generalist to specialist ratio had a mean of 1.06 (SD = 0.49), and the percentage of female physicians averaged 44.78% (SD = 13.21).

**Table 1:** Summary of descriptive statistics.

Variable	Mean	SD	Min	25th Percentile	Median	75th Percentile	Max
Democracy Index	5.611	2.099	0.32	4.23	6.41	7.12	9.93
Corruption Perception Index	41.10	20.26	18	25	43	58	88
Physician density)	24.45	14.32	2.54	14.30	20.00	35.00	58.58
Generalist to Specialist Ratio	1.06	0.49	0.17	0.70	1.00	1.38	2.71
% Female Physicians	44.78	13.21	18.30	36.75	45.10	52.15	72.40
Domestic Health Expenditure (% of GDP)	4.58	2.17	1.00	2.98	4.12	5.55	11.62

### Partial correlations

**Fig 1:**
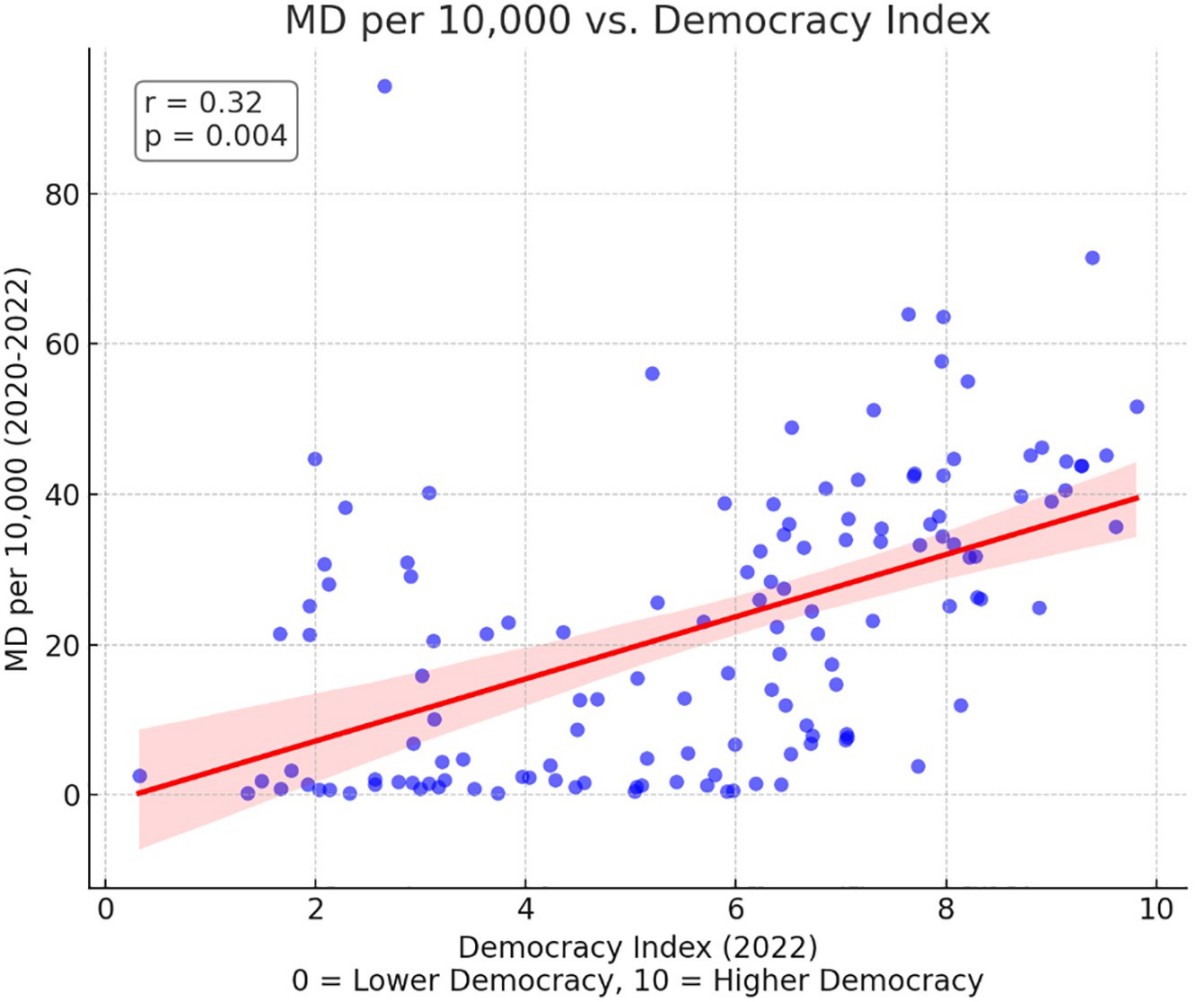
Correlation between democracy index and physician density (MD per 10,000).

**Fig 2:**
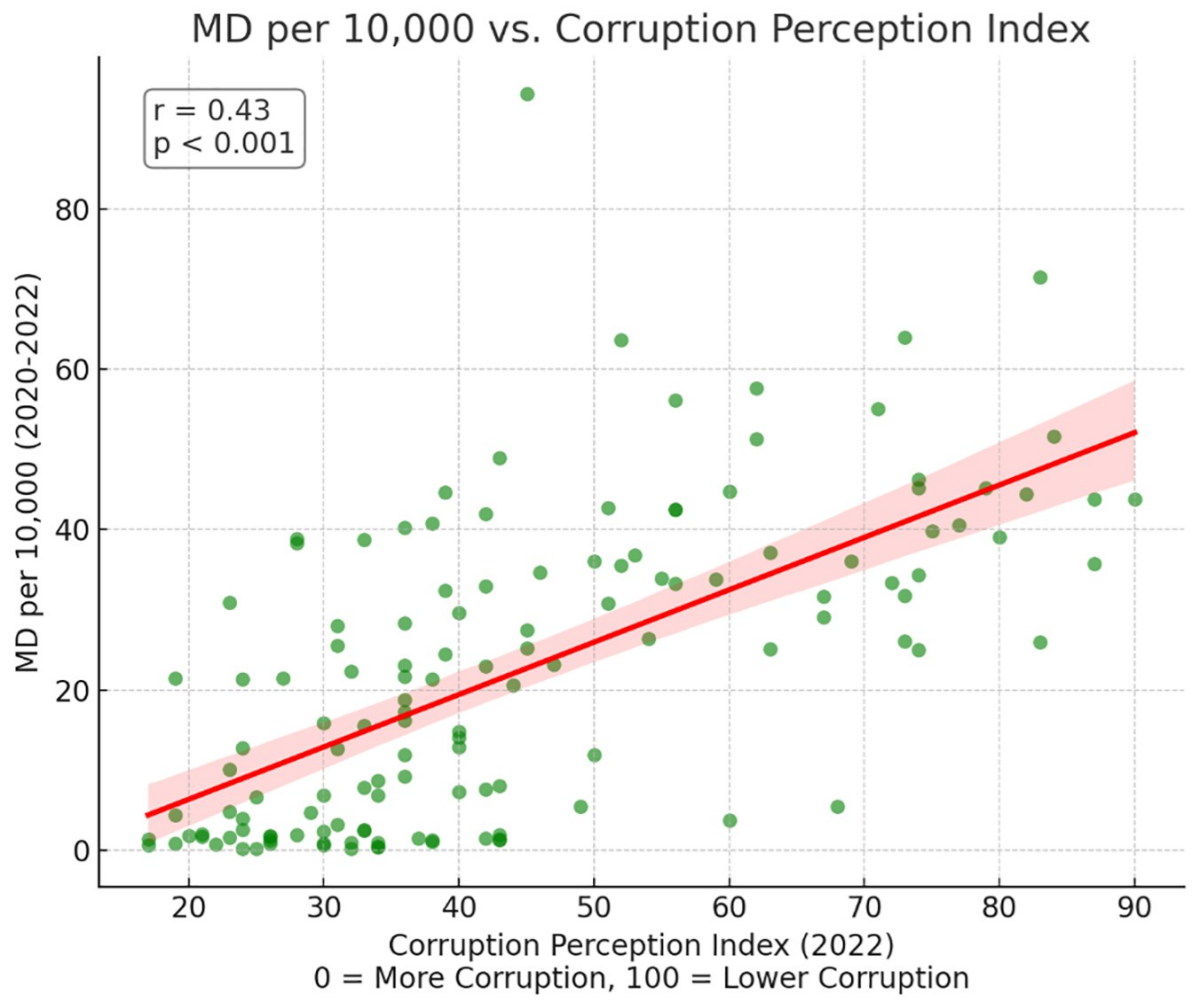
Correlation between corruption perception index and physician density (MD per 10,000).

Partial correlations results indicated significant positive correlations between DI and physician density (r = 0.32, p = 0.004) as well as between CPI and physician density (r = 0.43, p < 0.001) (Figs 1 and 2). These findings suggest that higher levels of democracy and lower levels of corruption are associated with greater physician density, independent of healthcare spending. However, no significant correlations were found between DI or CPI and the generalist to specialist ratio or the percentage of female physicians (Table 2). There was a positive correlation between domestic expenditure on healthcare and % female physicians (r = 0.377, p = 0.0002).

**Table 2:** Partial correlations.

Dependent Variable	Independent Variable	Correlation (r)	p-value
Physician density	Democracy Index	0.32	**0.004**
Physician density	Corruption Perception Index	0.43	**<0.001**
Generalist to Specialist Ratio	Democracy Index	0.05	0.661
Generalist to Specialist Ratio	Corruption Perception Index	0.13	0.246
% Female Physicians	Democracy Index	0.10	0.391
% Female Physicians	Corruption Perception Index	-0.03	0.789

### Multivariate Analysis of Variance (MANOVA)

MANOVA results indicated significant multivariate effects of both DI (Wilks’ Lambda = 0.8642, p = 0.013) and CPI (Wilks’ Lambda = 0.8036, p = 0.001) (Table 3). These findings suggest that governance indicators are collectively associated with healthcare workforce metrics.

**Table 3:** MANOVA results.

Independent Variable	Wilks’ Lambda	F	p-value
Democracy Index	0.8642	3.98	**0.013**
Corruption Perception Index	0.8036	6.29	**0.001**

### Univariate Analysis of Variance (ANOVA)

Univariate ANOVA results showed that both DI (F = 6.13, p = 0.015) and CPI (F = 10.57, p = 0.002) are significantly associated physician density, even after adjusting for domestic health expenditure (F = 18.53, p < 0.001) (Table 4). However, neither DI nor CPI had significant associations with the generalist to specialist ratio or the percentage of female physicians.

**Table 4:** Univariate ANOVA results.

Dependent Variable	Independent Variable	F-value	p-value
Physician density	Democracy Index	6.13	**0.015**
Physician density	Corruption Perception Index	10.57	**0.002**
Physician density	Domestic Expenditure	18.53	**<0.001**
Generalist to Specialist Ratio	Democracy Index	0.35	0.555
Generalist to Specialist Ratio	Corruption Perception Index	1.27	0.264
Generalist to Specialist Ratio	Domestic Expenditure	2.98	0.089
% Female Physicians	Democracy Index	2.14	0.148
% Female Physicians	Corruption Perception Index	0.64	0.426
% Female Physicians	Domestic Expenditure	**7.56**	**0.008**

## Discussion

The present study demonstrates that higher levels of democracy and lower levels of corruption are associated with a greater density of medical doctors, even when controlling for healthcare spending. While our findings extend the well-established link between governance quality and public health outcomes. [4, 5] to the healthcare workforce, they must be interpreted cautiously. The relationship between governance and health workforce outcomes is complex and may involve reverse causality, where stronger healthcare systems build public trust and governance capacity, just as good governance supports the workforce. Confounding factors such as economic development, historical challenges, political stability, and healthcare system efficiency may also play a role.

Although political stability was not included in this analysis, the benefits of democratic governance and reduced corruption likely extend to the availability of healthcare professionals, a crucial element of healthcare system capacity. These findings highlight the need for further exploration of how democracy and corruption independently and collectively shape the healthcare workforce.

Democratic institutions, by promoting political stability and inclusive policymaking, often lead to better investment in healthcare infrastructure and workforce development. For instance, South Korea’s democratic governance, characterized by transparency and public participation, was notably effective in managing its healthcare workforce amid political and economic challenges during the COVID-19 pandemic [13]. The country’s ability to rapidly mobilize resources and maintain public trust illustrates how key democratic principles, such as open communication, accountability, and collective decision-making, strengthened the healthcare system’s response. These democratic features fostered an environment where the healthcare workforce could be mobilized quickly and efficiently, helping to mitigate the effects of the pandemic [13–15].

The United Kingdom’s National Health Service (NHS) demonstrates how democratic systems, rooted in transparency and public scrutiny, directly influence workforce distribution and healthcare policy. In the UK, decisions regarding workforce planning and resource allocation are subject to democratic oversight, ensuring that the public is involved in debates over healthcare priorities [16]. The ongoing discussions around NHS workforce shortages, funding, and Brexit further reflect how democratic processes ensure that the healthcare workforce is shaped by public needs and accountability, despite the challenges posed by political shifts [17].

In contrast, the recent enactment of Indonesia’s Health Law No. 17 of 2023 highlights the consequences of low democratic engagement in healthcare decision-making. The law, which was passed with minimal public consultation and transparency, illustrates how limited democratic participation can undermine the healthcare workforce. The absence of stakeholder involvement and insufficient public input was one of many factors that eroded trust in the government’s ability to implement healthcare reforms, leading to concerns about healthcare workforce morale and retention [18]. This example underscores how the lack of democratic processes can weaken a country’s ability to maintain a robust healthcare workforce." [18].

These contrasting examples illustrate the how democratic systems, across geographic regions support transparency and public discourse and may in turn support the maintenance of the healthcare workforce. However, erosion of these systems may threaten access to healthcare providers in less democracy government structures.

Beyond systems of government, the significant association between Corruption Perception Index scores and physician density in our study suggests that abuse of power may have detrimental associations with the healthcare workforce [7, 8]. Real-work consequences of corruption on healthcare have been evident in Nigeria for example, which has a low physician density and high degree of corruption [19]. The diversion of public funds has resulted in a shortage of medical supplies, inadequate healthcare facilities, and a demoralized healthcare workforce, ultimately contributing to higher mortality rates and poor healthcare access [19]. Scholars have identified key aspects of the healthcare sector that make it particularly susceptible to corruption including uncertainty around the demand for services (i.e. knowing who will need care and when), involvement of private entities in public decision-making, and lack of transparency around public funds allocated to healthcare expenses [19, 20]. These examples align with our findings that corruption may deplete the healthcare workforce and anti-corruption efforts should be addressed within the larger mandate of UHC.

Our study found no significant associations between governance indicators and the generalist to specialist ratio or the percentage of female physicians. These results suggest that while democracy and corruption are significantly associated with the overall availability of medical doctors, they do not necessarily influence the specialty composition or gender distribution within the healthcare workforce. This may be due to systemic issues such as specific national policies or regional cultural factors that dictate the roles and specializations within the medical profession, independent of broader governance structures.

The findings of this study have significant implications for policymakers–including national governments and political leaders. National governments and political leaders are critical in promoting democracy and reducing corruption, both essential for achieving health-related goals. By fostering transparency, accountability, and public participation, governments can create environments that support effective healthcare systems, ensuring that resources are equitably allocated and healthcare policies serve the public interest [21]. Prioritizing anti-corruption measures, such as enforcing transparent procurement processes and strengthening legal frameworks, is vital for the efficient use of healthcare resources and the support of healthcare professionals [7, 22].

Our findings directly reinforce the World Health Organization’s (WHO) emphasis on good governance as essential for developing and sustaining a robust healthcare workforce. By demonstrating the significant associations between governance quality—specifically democracy and corruption—and healthcare workforce distribution, this study provides empirical support for the WHO’s recommendations on the importance of transparency, accountability, and public participation in strengthening health systems. These governance principles, as outlined by WHO, are crucial not only for improving health outcomes but also for ensuring an equitable distribution of healthcare professionals, which is foundational to achieving universal health coverage [23]. The WHO can further these efforts by providing technical support and advocating for governance reforms that align with health objectives, while also facilitating dialogue among member states to share best practices and promote collective action [24].

Our study has several limitations that must be acknowledged. While the cross-sectional design itself is not necessarily a limitation, the analysis methods chosen in this study limit the ability to fully explore potential causal relationships. The reliance on publicly available data may introduce biases, particularly in more corrupt or less democratic countries where data accuracy could be compromised [25]. Additionally, the study did not account for all potential confounding variables, such as specific health policies or cultural factors, that could influence the healthcare workforce. Moreover, our analysis included physician density, which, while important in supportive a robust health workforce, does not necessarily capture regional variation in workforce distribution within a country. Future research should consider using more complex statistical models, such as instrumental variables or longitudinal designs, to better address issues of causality and control for unmeasured confounders".

Future research should continue to explore the complex relationships between governance, health systems, and workforce dynamics using both quantitative and qualitative methods. Longitudinal studies could provide insights into how changes in political stability and corruption levels over time impact the shift in the healthcare workforce. Additionally, qualitative studies examining the experiences of healthcare workers in different governance contexts could offer valuable insights into the practical challenges and opportunities associated with various political environments [26].

Investigating the role of specific national policies, educational systems, and cultural factors in shaping the generalist to specialist ratio and the percentage of female physicians would also be beneficial. Such studies could help identify best practices and inform policy recommendations aimed at promoting a balanced and diverse healthcare workforce.

## Conclusion

Higher levels of democracy and lower levels of corruption are associated with greater physician density, highlighting the importance of political stability and transparency for effective health workforce planning and resource allocation. Policymakers must advocate for governance reforms that support a transparent and efficient health system and a robust healthcare workforce. By doing so, they can help bridge the gap between institutional efforts and broader governmental actions, ultimately supporting the goal of Universal Health Coverage.
